# Inclusion Complex of Isoliquiritigenin With Sulfobutyl Ether-β-Cyclodextrin: Preparation, Characterization, Inclusion Mode, Solubilization, and Stability

**DOI:** 10.3389/fchem.2022.930297

**Published:** 2022-06-21

**Authors:** Xiaozheng Wu, Jiamin Li, Chunmei Hu, Yingying Zheng, Yufei Zhang, Jianping Li, Mengyue Li, Di Xiao, Li Lu, Yuechang Huang, Xingmin Zhang, Chen Li

**Affiliations:** School of Biotechnology and Health Sciences, Wuyi University, Jiangmen, China

**Keywords:** isoliquiritigenin, sulfobutyl ether-β-cyclodextrin, inclusion complex, preparation, characterization

## Abstract

Isoliquiritigenin (ISL) possesses a wide variety of pharmacological properties, however, its poor solubility and oral bioavailability pose a significant barrier to its application. In present studies, the ISL inclusion complex was prepared with sulfobutyl ether-β-cyclodextrin (SBE-β-CD). The physicochemical characterizations of ISL-SBE-β-CD were performed with Fourier transform infrared (FT-IR) spectroscopy and X-ray powder diffraction (XRD). Phase solubility study suggested a 1:1 formation of ISL-SBE-β-CD complexes. The water solubility of ISL rose from 13.6 μM to 4.05 mM by the inclusion of SBE-β-CD. The antioxidant activities (IC50) of ISL-SBE-β-CD reached 42.2 μg/ml, which was significantly lower than that of ISL (60.5 μg/ml). Its stability in biological environments was also enhanced.

## Introduction

**FIGURE 1:**
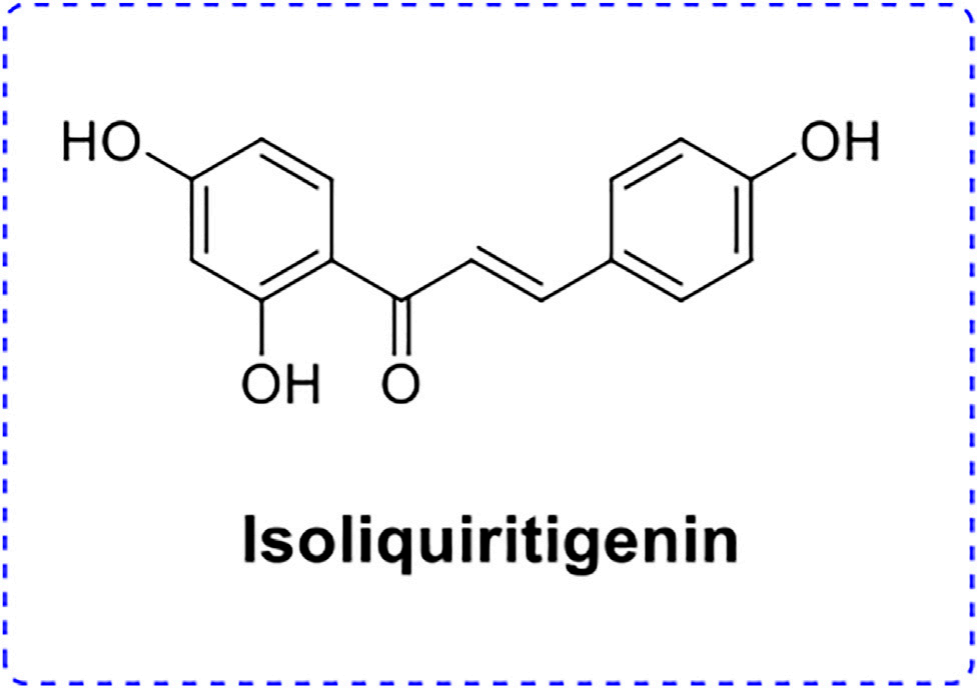
The chemical structure of isoliquiritigenin.

Isoliquiritigenin (ISL; Figure 1), a flavonoid, is the major bioactive constituent isolated from the food plants such as licorice, shallot, and bean sprouts (Cao et al., 2004; Cho et al., 2011; Tan et al., 2022). Numerous pharmacological studies have suggested that ISL exhibited various pharmacological properties including anti-inflammatory, antioxidant, analgesic, anticancer, antiplatelet aggregation, anti-angiogenic effect, and cytoprotective effects (Chin et al., 2007; Lee et al., 2009; Sun et al., 2022). Although many pharmacological activities of ISL have been recognized, its water solubility is relatively poor (Kakegawa et al., 1992; Kang et al., 2010). The poor water solubility of ISL would result in a slow dissolution and low absorption rate in the gastrointestinal tract, hence reducing oral bioavailability, which hinders its wide applications in pharmaceutical and functional foods (Jang et al., 2008). Microencapsulation is an effective method to maintain its bioactivity.

Cyclodextrins (CDs) are cyclic oligosaccharides equipped with a hydrophilic surface and a hydrophobic cavity, which can encapsulate hydrophobic molecules to prepare water-soluble complexes (Gotsev and Ivanov, 2009; Nguyen et al., 2013). Notably, when hydrophobic molecules were encapsulated in CDs, their biological activities were improved compared to free molecules (Fuchs et al., 1993; Irie and Uekama, 1997). Sulfobutylether-β-cyclodextrin (SBE-β-CD), a negatively charged derivative of β-CD, is prepared with SBE groups substituting the secondary hydroxyls of β-CD (Wu et al., 2013; Zhou et al., 2020). Hence, SBE-β-CD shows an extremely hydrophilic exterior surface and an extended hydrophobic cavity (Kucerova et al., 2016; Jafari et al., 2022). Especially, SBE-β-CD presents relatively low toxicity and high water-solubility than β-CD (Merzlikine et al., 2011; Shen et al., 2013). The formation of natural product molecules with inclusion complex with SBE-β-CD is of great interest in various fields (Fulop et al., 2015; Zhang et al., 2017; Zhao et al., 2020; Wang and Zhang, 2022). Although Li et al. (2015) reported the inclusion complex of isoliquiritigenin and 6-*o*-α-D-maltosyl-β-cyclodextrin. However, the ISL inclusion complex with SBE-β-CD has not been described.

Thus, the aim of the study is to investigate the inclusion complex of ISL with SBE-β-CD as a potential method to increase bioavailability. First, the interactions between the ISL and SBE-β-CD are determined by a phase solubility analysis. Then, the ISL-SBE-β-CD is characterized by Fourier transform infrared (FT-IR) spectroscopy and X-ray powder diffraction (XPRD). Finally, the bioavailability of the ISL complex with SBE-β-CD is evaluated.

## Experimental

### Instruments and Reagents

ISL was purchased from Xi’an Kai Lai Biological Engineering Co., Ltd. SBE-β-CD with an average molecular weight of 1,410 was provided by Nanjing Dulai Biotechnology Co., Ltd. All other reagents used were of analytical grade.

### Preparation of Isoliquiritigenin-Sulfobutyl Ether-β-Cyclodextrin Inclusion Complex

The ISL-SBE-β-CD inclusion complex was prepared by the method of an aqueous solution according to the previous method (Mohan et al., 2012; Mura, 2015). In brief, SBE-β-CD (6.50 mmol) was dissolved in 100 ml of distilled water at 60°C with continuous stirring for 1 h. The ISL solution containing 3.25 mmol of ISL in ethanol was added. Then the suspension solution was stirred at 60°C for 4 h. After removing ethanol, the solution was freeze-dried for 24 h and the ISL-SBE-β-CD inclusion complex was obtained.

### X-ray Powder Diffraction Assay

The X-ray powder diffraction patterns of ISL, SBE-β-CD, and ISL-SBE-β-CD were recorded on a Rigaku powder X-ray diffraction system (Siemens D5000, Germany). Powders were scanned at a diffraction angle of 2θ from 2 to 40°, with 40 KV and 30 mA under Cu Kα radiation (Qiu et al., 2014).

### Fourier Transform Infrared Spectroscopy Assay

The FT-IR spectra of ISL, SBE-β-CD, and ISL-SBE-β-CD were recorded using a Nicolet 6700 spectrophotometer (Thermo Fisher Scientific, United States). The FT-IR measurements of samples were performed in the scanning range of 4,000 cm^−1^ ∼400 cm^−1^ (Volobuef et al., 2012).

### Concentration–Absorbance Calibration Curve of Isoliquiritigenin

ISL (10 mg) was accurately weighed and dissolved into 100 ml ethanol–water solution (v: v = 1:1). ISL ethanol–water solution with concentrations of 1, 2, 3, 4, 5, 6, 7, 8, 9, and 10 μg/ml were prepared. The UV spectra of samples were measured with a UV-8000S from 200 to 600 nm. Also, then the concentration–absorbance calibration curve of ISL was made (Yang et al., 2009).

### Phase Solubility Study

Phase solubility study of ISL in the aqueous solution of SBE-β-CD was conducted according to the previous method (Chen et al., 2007; Aleem et al., 2008). An excess amount of ISL (8 mg) was added to 10 ml of SBE-β-CD solution with different concentrations (0–9 mM). The samples were shaken for 48 h at 25°C. After equilibrium, the samples were filtered through 0.22 μm PTFE filters to remove the excess ISL. Then, the UV absorption of ISL in each aqueous solution was determined at 374 nm, and the concentration of ISL in each aqueous solution was accounted for against the concentration–absorbance standard curve of ISL. The phase solubility profiles of ISL were obtained by plotting the solubility of ISL as a function of SBE-β-CD concentration.

The stability constants, *K*_c_, were calculated from the phase solubility diagram according to the Higuchi–Connors equation: *K*_c_ = slope/[S_o_×(1−slope)], where S_o_ is the ISL solubility in the absence of SBE-β-CD.

### Solubility Study

Solubility determination of pure ISL and ISL-SBE-β-CD was carried out in the aqueous solution according to the previous references. An access amount of ISL was added to a 5 ml aqueous solution, and then the suspension was shaken for 48 h at 25°C. After filtering through 0.22 μm PTFE filters, the UV absorption of ISL in each aqueous solution was determined at 374 nm, and the concentration was accounted for. The solubility of ISL-SBE-β-CD in an aqueous solution was measured using a similar method.

### Antioxidant Capacity

The DPPH radical scavenging assay was used to assay the antioxidant capacity (García-Padial et al., 2013). The samples were resolved in methanol and diluted to different concentrations, respectively. First, 1.5 ml sample solution was added to DPPH methanolic solution (1.5 ml, 0.1 mM) and laid in the dark for 30 min. Then, their absorbance was determined at 517 nm, respectively, and 50% inhibitory concentration (IC_50_) was obtained from the scavenging results.

## Results and Discussion

### X-ray Powder Diffraction Assay

**FIGURE 2:**
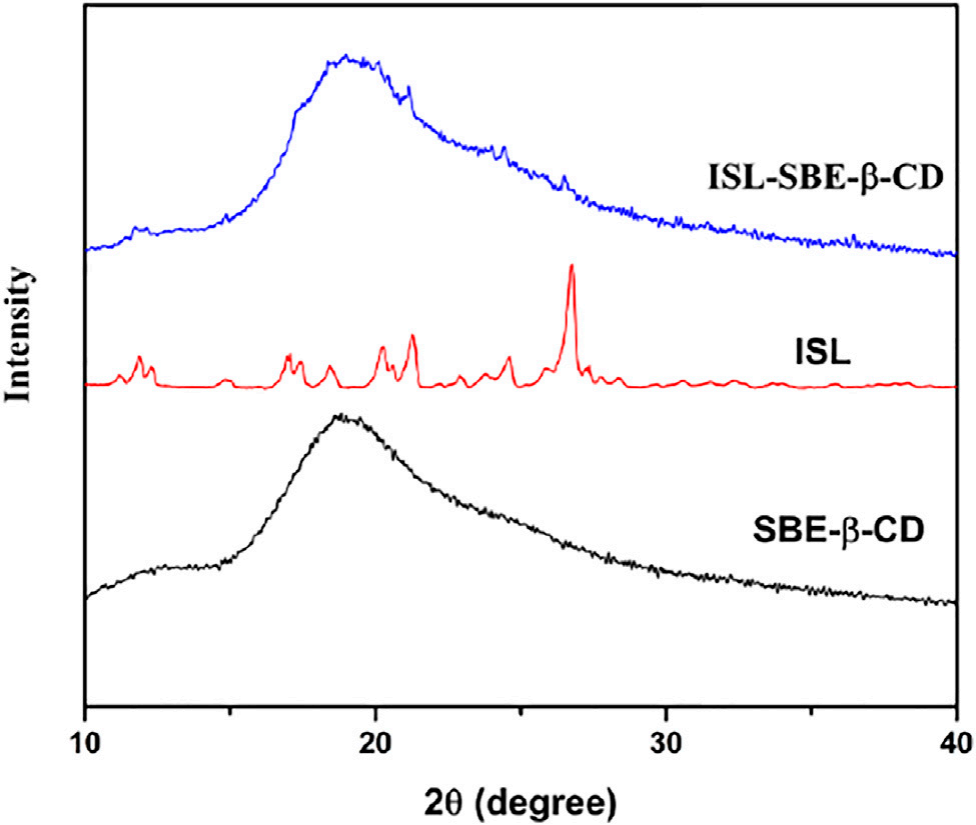
XRD patterns of ISL, SBE-β-CD, and ISL-SBE-β-CD.

The XRD spectra of ISL, SBE-β-CD, and ISL-SBE-β-CD are shown in Figure 2. The diffractogram of ISL showed various characteristic peaks at 12.3°, 17.1°, 18.4°, 20.2°, 21.3°, 24.6°, and 26.7°, indicating a highly crystalline structure. In contrast, the absence of characteristic peaks in the spectrum of SBE-β-CD reveals its amorphous state. Compared with ISL and SBE-β-CD, the diffraction of ISL-SBE-β-CD was similar to that of SBE-β-CD and showed no characteristic peaks of ISL. The XRD results showed that ISL was cocooned ion into the SBE-β-CD cavity, resulting in the self-lattice arrangement change of ISL from crystalline to amorphous state. These results were consistent with Li’s report (Li et al., 2015) that the inclusion complex form changed the self-lattice arrangement.

### Fourier Transform Infrared Assay

**FIGURE 3:**
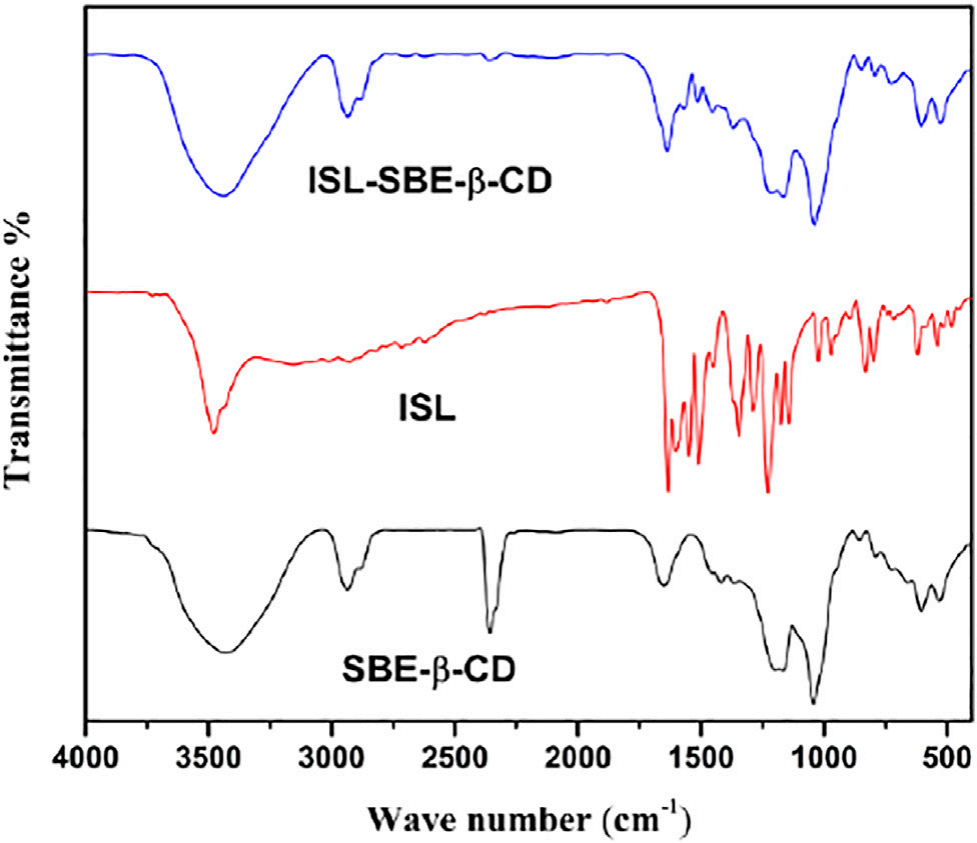
FT-IR spectra of ISL, SBE-β-CD, and ISL-SBE-β-CD.

The FT-IR spectra of ISL, SBE-β-CD, and ISL-SBE-β-CD are shown in Figure 3. The spectrum of ISL consisted of mainly significant groups including 3,479 cm^−1^ (O–H stretching vibration); 1,631 cm^−1^ (C=O group); and 1,600, 1,550, 1,510, and 1,450 cm^−1^ (aromatic nucleus). The FT-IR spectrum SBE-β-CD shows the main absorption bands at 3,490 cm^−1^ (O-H); 2,938cm^−1^ (C-H)^—^; 1,677cm^−1^ (O-H); and 1,217 and 1,050 cm^−1^ (C-H and C-O). But in FT-IR spectra of ISL-SBE-β-CD, its FT-IR spectra are close to that of SBE-β-CD, and the characteristic absorption band of ISL at 400–1600 cm^−1^ are overshadowed by corresponding ones of SBE-β-CD. These changes suggested that ISL got into the SBE-β-CD cavity and formed intra-molecular hydrogen bonds in the inclusion process of ISL and SBE-β-CD. The results were verified with XRD results.

### Concentration–Absorbance Calibration Curve of Isoliquiritigenin

**FIGURE 4:**
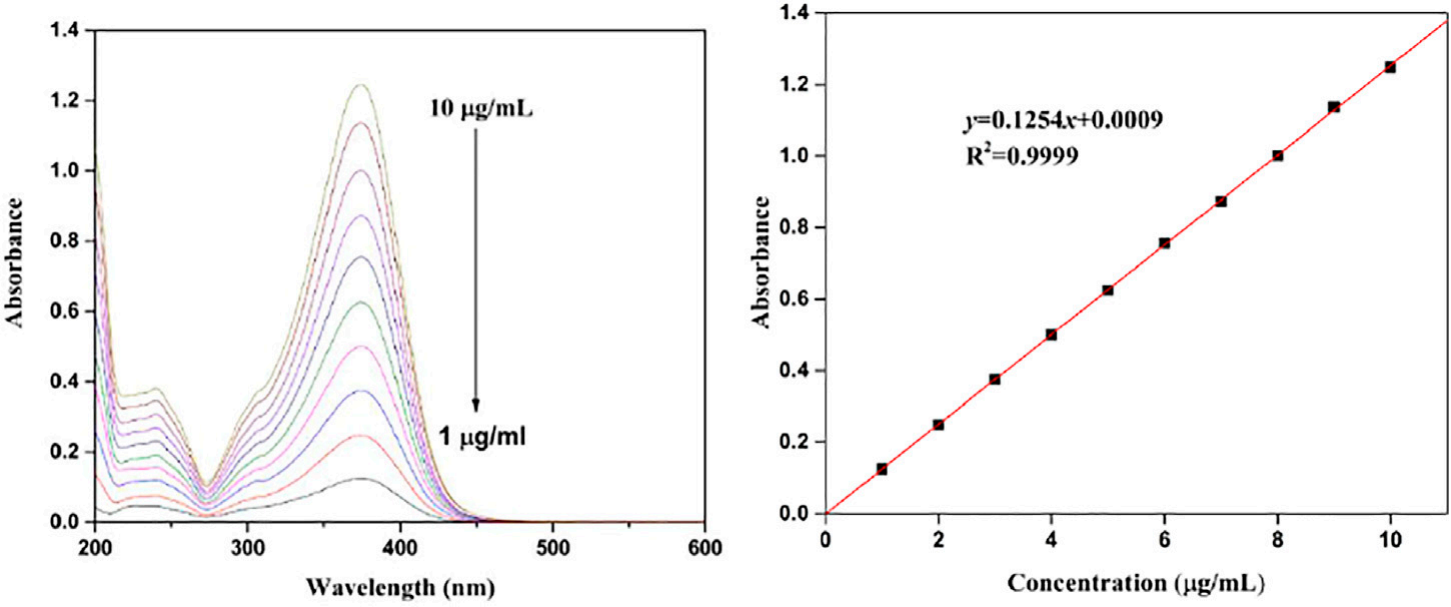
Concentration–absorbance calibration curve of ISL.

The UV spectra of ISL with different concentrations are illustrated in Figure 4. The characteristic absorption peak of ISL was found at 374 nm. The absorption intensity enhanced with the increase of ISL concentration, resulting in the concentration–absorbance calibration curve (*y* = 0.1254*x*+0.0009, R^2^ = 0.999).

### Phase Solubility Study

**FIGURE 5:**
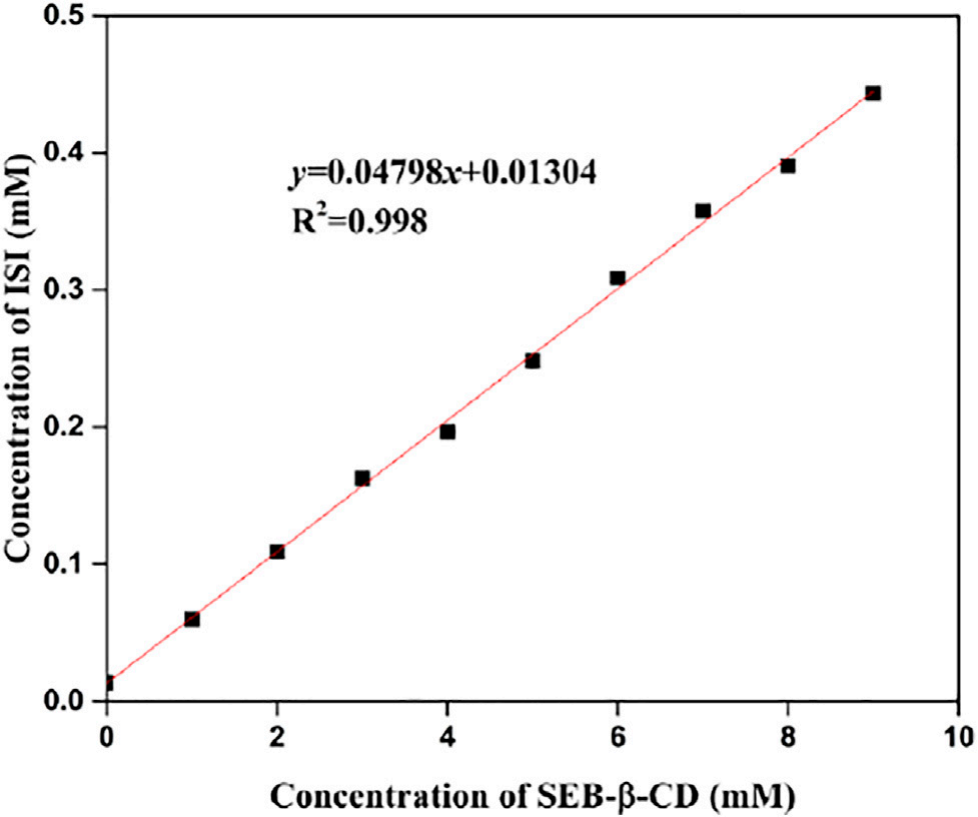
A phase solubility diagram of the ISL-SBE-β-CD system.

The phase solubility profiles of ISL-SBE-β-CD are presented in Figure 5. It could be seen that the concentration of ISL in water was obviously solubilized by the presence of SBE-β-CD. Moreover, the solubility curve was a straight line (R^2^ = 0.998). According to Higuchi and Connors, this profile could be described as an AL type, suggesting a 1:1 formation of ISL-SBE-β-CD complexes. The molar ratio of ISL to SBE-β-CD in this complex might be determined by the characteristics of ISL. Also, the stability constants, *K*c, of ISL-SBE-β-CD were calculated as 3864 M^−1^ according to the phase solubility diagram. The high *K*_c_ value verified a high tendency of ISL to enter the SBE-β-CD cavity.

### Effect of Sulfobutyl Ether-β-Cyclodextrin on Aqueous Solubility of Isoliquiritigenin

SBE-β-CD is usually used to enhance the aqueous solubility of drugs due to the large number of hydroxyl groups. SBE-β-CD could provide the lipophilic microenvironment for hydrophobic drug molecules. ISL was encapsulated into the hydrophobic cavity of SBE-β-CD interiors, leading to the form of non-covalent dynamic inclusion complexes. The aqueous solubility of ISL and ISL-SBE-β-CD was assessed. The water solubility of ISL was only 13.6 μM, while complexation led to a notable increase to 4.05 mM in water. A 298-fold increase in water solubility was achieved by the solubilizing effect of SBE-β-CD. Hence, SBE-β-CD could be used as an effective inclusion agent with a solubilizing effect.

### Stability in Biological Environments

To evaluate the stability of ISL-SBE-β-CD in biological environments, the absorbance changes of ISL and ISL-SBE-β-CD in a simulated gastric acid environment (ca. pH 1.5) and simulated intestinal fluid environment (ca. pH 7.6) were tracked. ISL and ISL-SBE-β-CD were dissolved in the buffer solution, respectively, and the absorbance at 374 nm was recorded. At pH 1.5 solution, the absorbance of ISL tapered off to 13.5 and 22.4% at 24 and 60 h, respectively. However, the absorbance of ISL-SBE-β-CD dwindled to only 6.8 and 14.2% at 24 and 60 h. At pH 7.6 solution, the absorbance of ISL and ISL-SBE-β-CD was similarly changed before 60 h, tapered off to 95.3 and 96.8%. These results showed that ISL-SBE-β-CD was more stable than ISL at both pH 1.5 and pH 7.6. Thence, SBE-β-CD could improve the stability of the included compounds.

### Antioxidant Capacity

**FIGURE 6:**
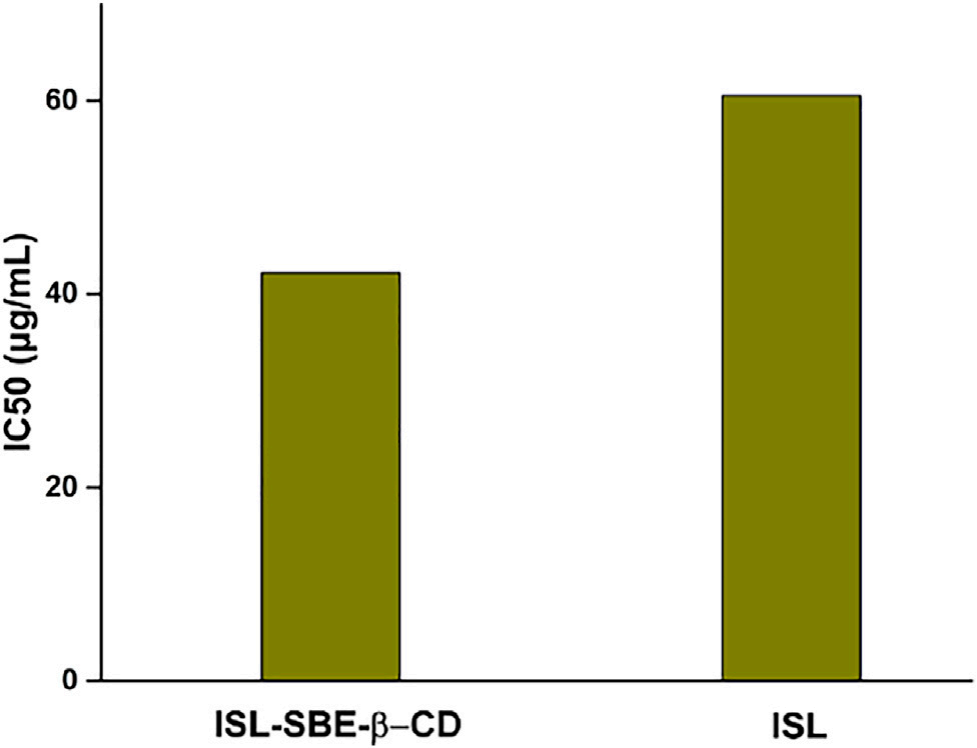
Antioxidant activities of ISL and ISL-SBE-β-CD system.

The DPPH assay results of ISL and ISL-SBE-β-CD are shown in Figure 6. The antioxidant activity (IC_50_) of ISL-SBE-β-CD was 42.2 μg/ml, which was significantly higher than that of ISL (60.5 μg/ml). The results showed that the ISL-SBE-β-CD presented a much higher antioxidant capacity than ISL. Thence, the antioxidant capacity of compounds can be optimized by the presence of cyclodextrins.

## Conclusion

The inclusion complexation behavior and characterization of isoliquiritigenin with sulfobutyl ether-β-cyclodextrin were investigated. The results showed that sulfobutyl ether-β-cyclodextrin could enhance the water solubility and stability of isoliquiritigenin. Moreover, inclusion complexation presented a much higher antioxidant capacity than isoliquiritigenin. Given the shortage of applications for isoliquiritigenin, the inclusion complexation would be regarded as an effective step in the development of a novel formulation of isoliquiritigenin for medicine or healthcare products.

## Data Availability

The original contributions presented in the study are included in the article/Supplementary Material; further inquiries can be directed to the corresponding author.
